# Beneficial Effect of Moderate Exercise in Kidney of Rat after Chronic Consumption of Cola Drinks

**DOI:** 10.1371/journal.pone.0152461

**Published:** 2016-03-31

**Authors:** Gabriel Cao, Julián González, Angélica Müller, Graciela Ottaviano, Giuseppe Ambrosio, Jorge E. Toblli, José Milei

**Affiliations:** 1 Instituto de Investigaciones Cardiológicas “Prof. Dr. Alberto C. Taquini”, ININCA-UBA-CONICET, Buenos Aires, Argentina; 2 Division of Cardiology, University of Perugia School of Medicine, Perugia, Italy; Emory University, UNITED STATES

## Abstract

**Aim:**

The purpose of this study was to investigate the effect of moderate intensity exercise on kidney in an animal model of high consumption of cola soft drinks.

**Methods:**

Forty-eight *Wistar Kyoto* rats (age: 16 weeks; weight: 350–400 g) were assigned to the following groups: WR (water runners) drank water and submitted to aerobic exercise; CR (cola runners) drank cola and submitted to aerobic exercise; WS (water sedentary) and CS (cola sedentary), not exercised groups. The aerobic exercise was performed for 5 days per week throughout the study (24 weeks) and the exercise intensity was gradually increased during the first 8 weeks until it reached 20 meters / minute for 30 minutes. Body weight, lipid profile, glycemia, plasma creatinine levels, atherogenic index of plasma (AIP) and systolic blood pressure (SBP) were determined. After 6 months all rats were sacrificed. A kidney histopathological score was obtained using a semiquantitative scale. Glomerular size and glomerulosclerosis were estimated by point-counting. The oxidative stress and pro-inflammatory status were explored by immunohistochemistry. A one way analysis of variance (ANOVA) with Tukey-Kramer post-hoc test or the Kruskal-Wallis test with Dunn’s post-hoc test was used for statistics. A value of p < 0.05 was considered significant.

**Results:**

At 6 months, an increased consumption of cola soft drink was shown in CS and CR compared with water consumers (p<0.0001). Chronic cola consumption was associated with increased plasma triglycerides, AIP, heart rate, histopathological score, glomerulosclerosis, oxidative stress and pro-inflammatory status. On the other hand, moderate exercise prevented these findings. No difference was observed in the body weight, SBP, glycemia, cholesterol and plasma creatinine levels across experimental groups.

**Conclusions:**

This study warns about the consequences of chronic consumption of cola drinks on lipid metabolism, especially regarding renal health. Additionally, these findings emphasize the protective role of exercise training on renal damage.

## Introduction

Chronic kidney disease (CKD) is associated with a high incidence of cardiovascular morbidity and mortality, due to renal dysfunction correlates with a striking increase in cardiovascular events. Undoubtedly, soft drinks are a major source of added sugar worldwide, and their consumption has been linked to obesity, diabetes, and metabolic syndrome [1–3]. Epidemiological and experimental evidence indicate that a greater consumption of sweet carbonated beverages is associated with overweight and obesity by virtue of the high sugar content, low satiety, and incomplete compensation for total energy in subsequent meals [4]. Hyperlipidemia is associated with CKD as proposed by the lipid nephrotoxicity hypothesis, suggesting that glomerulosclerosis and atherosclerosis may share similar pathophysiological mechanisms linked to hyperlipidemia. Chronic consumption of cola drinks causes alterations in lipid profile of rats and *APO-E mice* [5–7], which includes an increase in plasma triglyceride levels. In addition, epidemiological studies indicate that high consumption of sugar-sweetened beverages increases the risk of CKD. In line with this, Samuelsson et al. [8] demonstrated a strong correlation between triglyceride-rich apoB-containing lipoproteins and the rate of progression in nondiabetic patients with CKD. In addition, Muntner et al. [9] showed that people with low HDL cholesterol and hypertriglyceridemia at baseline have a higher risk for having a loss of renal function. In agreement with this statement, high triglycerides level is considered an independent predictor of renal disease, as it was confirmed in a prospective study of 297 patients with type 1 diabetes [10]. Furthermore, the atherogenic index of plasma (AIP) was also proposed as a marker of plasma atherogenesis because it is increased in people at higher risk for coronary heart disease [11].

Several studies have suggested that renal tubular epithelial cells play a pro-inflammatory role by releasing some important mediators of acute and chronic renal injury, such as interleukin 6 (IL6) and Tumor Necrosis Factor alpha (TNFα) [12, 13].

It is mostly accepted that the principal pathological pathway that connects oxidative stress, inflammation, and CKD progression is characterized by an original injury in the kidney as a consequence of oxygen-derived radicals and the consequent inflammatory reaction. On the other hand, thioredoxin-1 (trx1) and peroxiredoxin-1 (prx1), an ubiquitous family of cysteine-dependent antioxidant proteins present in mammalian cells, representing a protective system against renal tubulointerstitial fibrosis induced by oxidative stress [14, 15].

Notably, a number of studies have demonstrated that regular physical activity prevents type 2 diabetes, cardiovascular disease [16], and premature mortality [17] because aerobic exercise can adaptively decrease the incidence of oxidative stress-associated diseases [18–20]. Therefore, the purpose of this study was to investigate the effect of moderate intensity exercise on kidney health following chronic consumption of cola soft drinks and the role of the tubular epithelial cells in releasing proinflammatory mediators that could participate in the renal damage.

## Material and Methods

The animal handling, maintenance and euthanasia procedures were performed according to international recommendations [21].

All animal experiments were approved by the Committee of Ethics in Animal Research of the Instituto de Investigaciones Cardiológicas (ININCA) and the Institutional Animal Care and Use Committee (IACUC) of the Faculty of Medicine of the University of Buenos Aires (CICUAL, Institutional Committee for the Care and Use of Laboratory Animals).

### Experimental design

Forty-eight adult male *Wistar-Kyoto* rats (age: 16 weeks; weight: 350–400 g) were randomly assigned to 4 groups (12 animals per group), receiving 2 different beverages ad libitum as the only liquid source for 6 months: water and regular cola (commercially available sucrose-sweetened carbonated drink, Coca-Cola^™^, Argentina). In addition, half of rats that consumed water or cola soft drink were submitted to aerobic exercise, constituting the following experimental groups: WR (water runners), WS (water sedentary), CR (cola runners), and CS (cola sedentary).

The aerobic exercise was performed for 5 days per week throughout the study and consisted of running on incline up to 10° on a treadmill Columbus^™^ designed for simultaneous practice of 4 animals. Exercise intensity (running speed and time) gradually was increased during the first 8 weeks until it reached 20 meters / minute for 30 minutes, as detailed below [22, 23] (Table 1).

**Table 1:** Exercise protocol.

Variable	Measure	Wk 1	Wk 2	Wk 3	Wk 4	Wk 5	Wk 6	Wk 7	Wk 8	Wks 9 to 24
**Speed**	**Meters/Min**	5	7	9	11	13	15	18	20	20
**Incline**	**Degrees**	10	10	10	10	10	10	10	10	10
**Duration**	**Minutes**	4	8	12	16	20	24	27	30	30
**Frecuency**	**Sessions/Wk**	5	5	5	5	5	5	5	5	5
**Distance per session**	**Meters**	20	56	108	176	260	360	486	600	600
**Distance per week**	**Meters**	100	280	540	880	1300	1800	2430	3000	3000

Six months after the beginning of the study (end of the experiment), the animals in each group (WR, WS, CR and CS) were euthanized by subtotal exsanguination under anesthesia (sodium thiopental 40 mg/kg, i.p.).

In order to minimize carbon dioxide content in the soft drinks, a vigorous stirring using a stirring plate and placing a magnetic bar in a container filled with the liquid at room temperature was performed prior to offering the beverage to the animals. One group that drank cola soft drink or water, were submitted to exercise as detailed below.

According to the company specifications, Coca Cola^™^ is a carbonated water solution containing (for each 100 ml; energetic value 43 Kcal): carbohydrate 10.6 g, sodium 7 mg, caffeine 11.5 mg, caramel, phosphoric acid, citric acid, vanilla extract, natural flavorings (orange, lemon, nutmeg, cinnamon, coriander, etc.), lime juice and fluid extract of coca (*Erythroxylon novogranatense*). Rats were weighed weekly. In order to assess the mean consumption of food and drink by 24 hours throughout seven days, the following procedure was performed. At the beginning of the experiment (07.00AM), feeders and drinking bottles were replaced by others with a known volume of food and drink. The remnant quantity of food and drink was also measured. This procedure was used twice a week in random days and, in the five running days, daily.

Biochemical determinations and systolic blood pressure (SBP) measurements were performed at baseline, 3 and 6 month of the experiment; histopathological data were obtained at the time of sacrifice (6 month). Animals were supplied and housed at the ININCA facilities under controlled temperature (21 ± 20 C) and 12-h light-dark cycles (7 am to 7 pm). Rats fed a commercial chow (16%-18% protein, 0.2 g % sodium; Cooperación, Buenos Aires, Argentina) *ad libitum*.

### Biochemical and blood pressure evaluations

At baseline, 3 and 6 months, plasma levels of glucose, total cholesterol, LDL-C, HDL-C and triglycerides (TG) were determined by enzymatic conventional assays in blood samples collected from the tail vein after 4-h fasting [24] and expressed in mmol / l. The atherogenic index of plasma (AIP) was calculated according to the following equation [11]:

AIP = log(TG/HDL−C)

The renal function considered in this study was defined as: serum creatinine at 3 and 6 month, and Δ creatinine (Δ Cr) (the change in creatinine from 3 to 6 months expressed in μmol/l) [25]. SBP was measured by tail cuff plethysmography in unanesthetized rats restrained in a plastic chamber. The average of at least 3 readings per session was recorded. A pneumatic pulse transducer positioned on the ventral surface of the tail, distal to the occlusion cuff, detected the return of the pulse following a slow deflation of the cuff. Cuff pressure was determined by a pneumatic pulse transducer, using a programmed electro-sphygmomanometer PE-300 (Narco Bio-Systems, Austin, Texas). Pulses were measured weekly during light cycle on a Physiograph MK-IIIS (Narco Bio-Systems, Austin, Texas) and the monthly average of heart rate (HR) was recorded.

### Tissue processing and morphological analysis

After animal sacrifice, kidneys were removed, perfused with saline solution through the renal vein, weighed, cut longitudinally and fixed in phosphate buffered 10% formaldehyde (pH 7.2). The resulting tissue pieces were embedded in paraffin, sectioned (4 μm thick), and stained with hematoxylin and eosin, periodic acid-Schiff (PAS) and Masson trichrome (MT) according to routine histological staining. The tissues were examined without knowledge of the experimental groups. The kidney sections were graded based on the presence and severity of abnormalities in glomeruli, tubules, vessels and interstitium using a semiquantitative scale. An overall histopathological score for each kidney was obtained and expressed in percentage [26].

The glomerular volume (Vg) was estimated by the maximal profile area (MPA) method and expressed in 10^6^μm^3^ [26]. For the MPA analysis, was employed the point-counting method using an orthogonal grid with 300 test points, representing an area of 6.7 10^4^μm^2^ at 40X objective lens, projected onto the fields of view. The number of points hitting the glomeruli was counted in at least 50 glomerular profiles per kidney. Thus, the MPA was expressed as follows [27]:

$$MPA\;\left(\mu m^{2}\right)\;=\;n\;x\;d^{2}$$

where n represents the number of points hitting the glomerular profile and d^2^ is the square of the distance between grid points. The glomerulosclerosis, estimated by point-counting method and expressed in percentage was defined by the presence of focal and segmental glomerular scarring and obliteration of glomerular capillaries with increased mesangial cellularity, mesangial matrix expansion, and adhesion formation between the tuft and Bowmans’ capsule.

### Immunohistochemistry

Paraffin-embedded sections were subjected to immunohistochemical assays as reported previously [28]. Briefly, the sections were deparaffinized with xylene, rehydrated through graded series of ethanol to water, and then incubated in blocking solution (PBS + 1% bovine serum) at room temperature for 1 h. Sections were then incubated overnight at 4°C with one of the following primary antibodies: rabbit polyclonal anti-trx1 (1:200 dilution; Abcam, Cambridge, MA), rabbit polyclonal anti-prx1 (1:200 dilution; Abcam, Cambridge, MA), goat polyclonal anti-IL-6 (1:100 dilution; Santa Cruz Biotechnology, Santa Cruz, CA) and monoclonal antibody against rat TNF-α (1:50 dilution; R&D Systems, Minneapolis, MN). All antibodies were diluted with blocking solution. Immunostaining was carried out with an avidin-biotin-peroxidase complex kit, counterstaining with hematoxylin and expressed in integrated optical density (IOD) units [29]. The tubular staining for trx1 (T_trx1_), prx1 (T_prx1_), IL6 (T_IL6_) and TNFα (T_TNFα_) was evaluated through an image processing software (Image-Pro Plus version 6; Media Cybernetics, Silver Spring, Maryland, USA). Control sections were incubated with non-immune normal rabbit serum.

### Statistical methods

Values were expressed as mean±SD. All statistical analyses were based on absolute values and processed by GraphPad Prism version 5.0 (GraphPad Software, Inc, San Diego, California, USA). Normal distribution was assessed by the Kolmogorov and Smirnov method with an assumption test. For parameters with a normal distribution such as the biochemical data, the groups were compared by a one-way analysis of variance (ANOVA) with Tukey-Kramer post-hoc test. HR of experimental groups was compared by a one-way analysis of variance (ANOVA) with Tukey-Kramer post-hoc test. Parameters such as histological scores or stereological data with non-normal distribution analyzed by the Kruskal-Wallis test with Dunn’s post-hoc test. A value of p<0.05 was considered significant.

## Results

### General findings

After cola consumption, the animals showed reduction in food intake (CS: 29.9±2.1 g/kg/24 hs; CR: 28.5±2.6 g/kg/24 hs) and increased fluid intake (CS: 82.2±12.6 ml/kg/24 hs; CR: 99.8±14.2 ml/kg/24 hs) compared with water consumers (p<0.05). Notably, increased plasma levels of TG and AIP (CS: 3.4±0.8 mmol/l and 0.6±0.1; CR: 3.7±2.6 mmol/l and 0.5±0.6, respectively) were shown in chronic cola drinkers. Furthermore, a slow increase of ΔCr along the experiment was observed in WR rats (CS: 12.2±4.8 μmol/l; CR: 11.3±11.8 μmol/l; WS: 11.2±13.2 μmol/l; WR: 3.7±6.4 μmol/l; p<0.05) (Table 2).

**Table 2:** Body weight, systemic blood pressure and laboratory findings of the experimental groups.

VARIABLE	CS	CR	WS	WR	p
**Body weight (g)**	712.3±88.7	715.6±56.9	693.9±76.7	673.2±39.8	NS
**SBP (mmHg)**	138.1±22.5	139.3±8.8	131.6±17.9	138.0±9.4	NS
**Food intake (g/kg/24 hs)**	29.9±2.1*	28.5±2.6*	36.7±5.5	38.7±5.8	<0.05
**Fluid intake (ml/kg/24 hs)**	82.2±12.6**	99.8±14.2*	60.1±4.5	74.7±9.6	<0.05
**Glucose (mmol/l)**	7.3±0.8	7.7±1.2	6.6±1.3	7.5±1.1	NS
**Δ Cr (μmol/l)**	12.2±4.8	11.3±11.8	11.2±13.2	3.7±6.4***	<0.05
**Total Cholesterol (mmol/l)**	3.4±0.8	3.5±1.0	3.4±1.0	3.1±0.7	NS
**LDL-C (mmol/l)**	1.2±0.4	1.2±0.4	1.47±0.5	1.3±0.4	NS
**HDL-C (mmol/l)**	0.9±0.2	0.9±0.2	0.8±0.2	0.8±0.2	NS
**TG (mmol/l)**	3.4±0.8*	3.7±2.6*	2.0±1.4	1.7±0.8	<0.05
**AIP (log[TG/HDL-C])**	0.6±0.1*	0.5±0.6*	0.3±0.2	0.3±0.1	<0.05

Values are means±SD and were analyzed using the one-way ANOVA with Tukey-Kramer post-hoc test. A value of p <0.05 was considered significant.

* vs WS and WR.

** vs CR and WS.

*** vs CS, CR and WS

SBP: systemic blood pressure; TG: triglycerides; AIP: atherogenic index of plasma.

Experimental Groups: CS: cola sedentary; CR: cola runners; WS: water sedentary; WR: water runners.

No significant difference was observed in the creatinine levels at baseline, 3 and 6 months, as shown in Table 3.

**Table 3:** Plasma creatinine levels at baseline, 3 and 6 months.

Experimental Time	CS _Cr_ (μmol/L)	CR _Cr_ (μmol/L)	WS _Cr_ (μmol/L)	WR _Cr_ (μmol/L)	p—value
Baseline	45.12±3.21	45.61±3.52	46.02±2.56	46.13±4.62	NS
3 month	45.88±3.19	46.77±5.75	47.81±2.91	49.26±6.25	NS
6 month	58.08±4.79	58.02±12.82	59.01±13.67	52.96±2.72	NS

Values are means±SD and compared by a one-way analysis of variance (ANOVA) with Tukey-Kramer post-hoc test.

Experimental Groups: CS: cola sedentary; CR: cola runners; WS: water sedentary; WR: water runners.

At the 3rd month, a progressive increase of heart rate in the CR rats was observed when compared to the WR (CR: 542±26.4 to 653±35.2 beats / min; WR: 521±27.5 to 573±26.2 beats / min), as illustrated in Table 4. A higher heart rate was also observed in CR versus in CS (CR: 622±34.2 and 653±35.2 beats / min; CS: 592±25.1 and 603±23.7 beats / min) at 5 and 6 months (Table 4). Likewise, from 3 months, WR showed a heart rate reduction compared with WS (Table 4).

**Table 4:** Heart rate of WS, WR, CS and CR experimental groups.

Months	WS (beats / min)		WR (beats / min)		CS (beats / min)		CR (beats / min)		p
	N	Mean±SD	N	Mean±SD	N	Mean±SD	N	Mean±SD	
0	48	371±23.2	48	367±27.3	48	360±25.2	48	361±26.2	NS
1	48	439±23.4	48	448±24.1	48	442±24.5	48	451±25.1	NS
2	48	472±24.5	48	466±24.3	48	464±24.8	48	470±25.0	NS
3	48	534±22.3	48	521±27.5*	48	532±23.6	48	542±26.4	<0.05
4	48	557±22.8	48	544±25.4*	48	564±24.2	48	574±31.1**	<0.05
5	48	582±24.3	48	567±27.1*	48	592±25.1	48	622±34.2***	<0.05
6	48	593±23.4	48	573±26.2*	48	603±23.7	48	653±35.2***	<0.05

Values are means±SD and were analyzed using the one-way ANOVA with Tukey-Kramer post-hoc test. A value of p <0.05 was considered significant.

* vs WS, CS and CR.

** vs WS.

*** vs WS and CS.

Experimental Groups: WS: water sedentary; WR: water runners; CS: cola sedentary; CR: cola runners.

No difference was observed in the body weight, SBP, glycemia and plasma cholesterol levels among experimental groups.

### Morphological changes at 6 month

**Fig 1:**
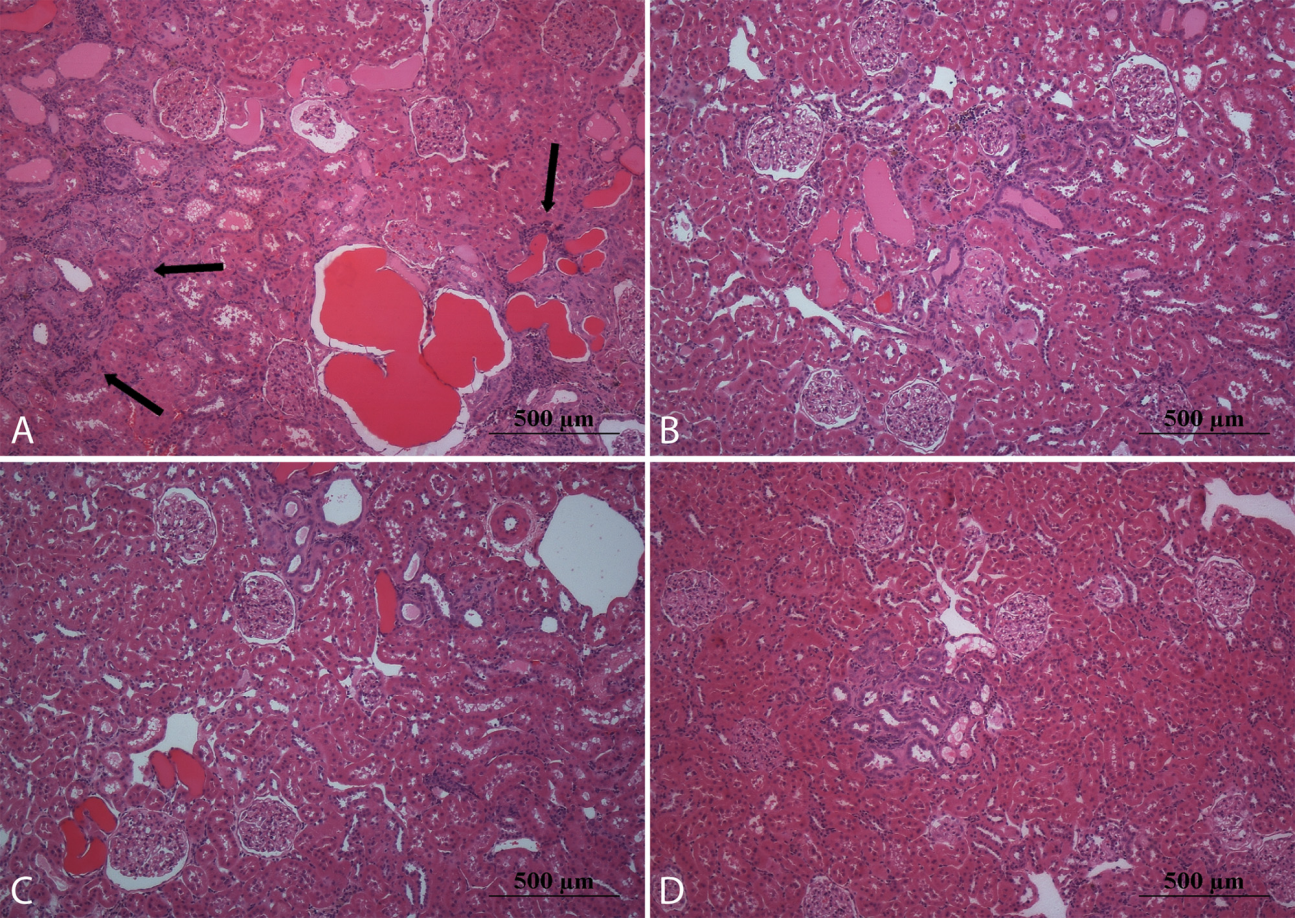
Representative microphotographs of kidney stained with hematoxylinand eosin. In sedentary rats that consume cola soft drink (A) were observed glomerular hypertrophy, glomerulosclerosis, tubular atrophy and dilatation, tubular casts, interstitial fibrosis and chronic inflammatory infiltrates (black arrows). The moderate exercise regimen partially protects against chronic consumption of cola drinks (B). These lesions were less apparent in rats that consume water (C and D). Magnification: 100X, scale bar 500 microns.

**Fig 2:**
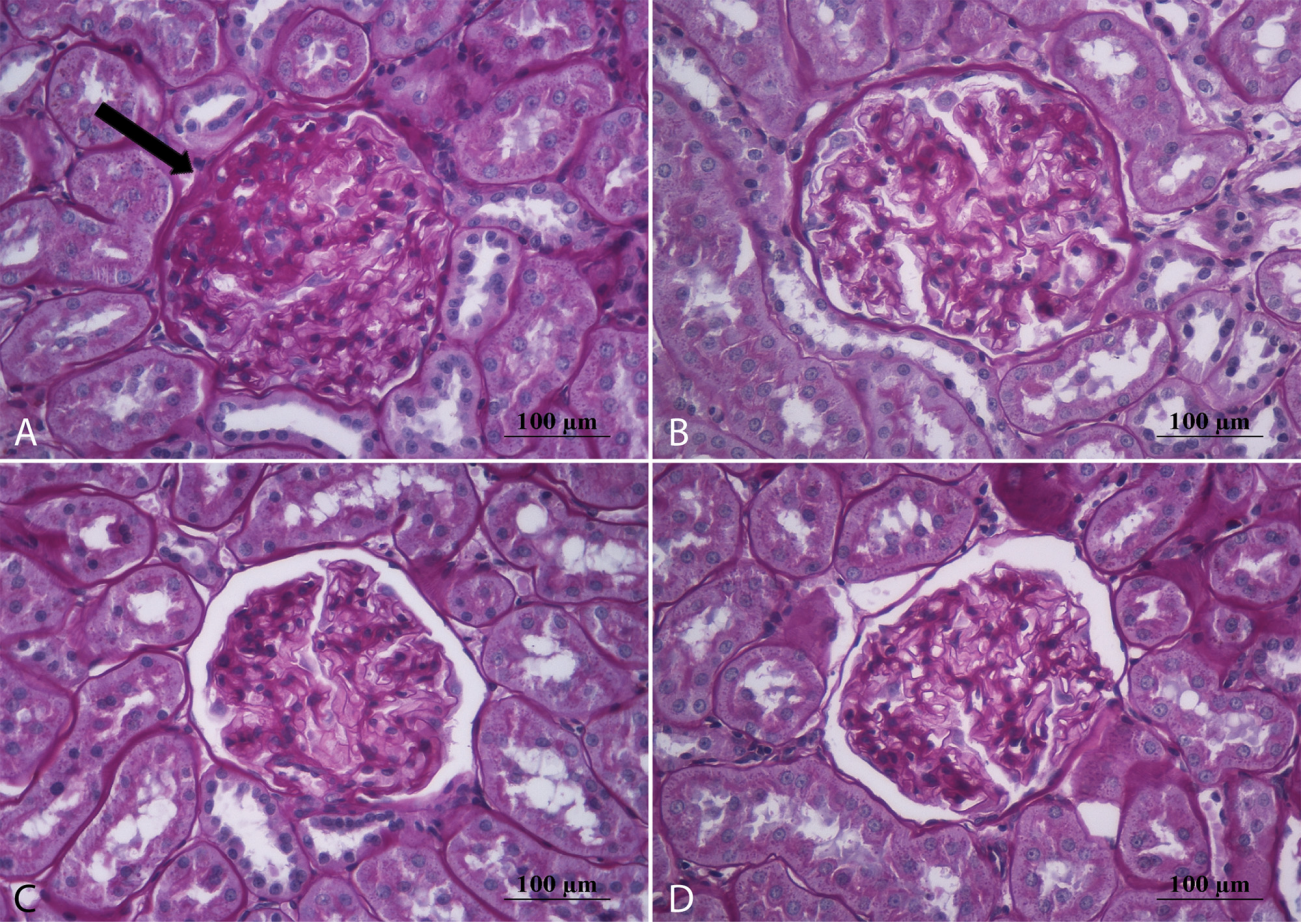
Representative microphotographs of glomeruli stained with periodic acid-Schiff. Sedentary rats that consume cola soft drink (A) showed segmental scarring and obliteration of glomerular capillaries with increased mesangial cellularity, mesangial matrix expansion and adhesion formation between the tuft and Bowmans’ capsule (black arrow). These pathological changes, corresponding to glomerulosclerosis, werelower in CR (B), WS (C) and WR (D) groups. Magnification: 400X, scale bar 100 microns.

Although sedentarism was associated with an elevated histopathological score regardless of the drink consumed (CS: 10.4±1.5; WS: 9.2±1.3), it is noteworthy, the WS group showed better histopathological score than CS (p<0.05). On the other hand, a decreased in Vg was observed in WR (1.4±0.5 10^6^μm^3^) compared to the other experimental groups (CS: 1.6±0.6 10^6^μm^3^; CR: 1.7±0.6 106μm3; WS: 1.7±0.6 10^6^μm^3^, p<0.05) (Table 4 and Fig 1). Both chronic consumption of cola soft drink and sedentarism were associated with elevated glomerulosclerosis in CS (26.2±10.3%) compared to CR (22.2±8.3%), WS (20.5±7.4%) and WR (21.9±7.6%) (p<0.05). (Table 5 and Fig 2).

**Table 5:** Morphological and immunohistochemical data of experimental groups.

VARIABLE	CS	CR	WS	WR	p
**Histopathological score**	10.4±1.5*	6.2±0.9	9.2±1.3*	5.2±1.1	<0.05
**Vg (10**^**6**^**μm**^**3**^**)**	1.6±0.6	1.7±0.6	1.7±0.6	1.4±0.5**	<0.05
**Glomerular sclerosis (%)**	26.2±10.3***	22.2±8.3	20.5±7.4	21.9±7.6	<0.05
**T**_**trx1**_ **(IOD)**	5.8±1.9*	10.8±3.6	6.9±4.5*	19.8±7.3	<0.05
**T**_**prx1**_ **(IOD)**	6.5±2.2*	11.8±4.2	5.6±2.3*	21.2±6	<0.05
**T**_**IL6**_ **(IOD)**	25.5±7.7*	4.5±1.5	3.3±1.1	1.4±0.3	<0.05
**T**_**TNFα**_ **(IOD)**	18.3±7.9*	5.9±2.3	3.8±1.2	1.6±0.4	<0.05

Values are means±SD and were analyzed using the Kruskal-Wallis test with Dunn´s post-hoc test. A value of p <0.05 was considered significant.

* vs CR and WR.

** vs CS, CR and WR.

*** vs CR, WS and WR.

Vg: glomerular volume; T_trx1_, T_prx1_, T_IL6_ and T_TNFα_: tubular immunostaining for thioredoxin-1, peroxiredoxin-1, interleukin-6 and tumor necrosis factor alpha, respectively; IOD: integrated optical density units.

Experimental Groups: CS: cola sedentary; CR: cola runners; WS: water sedentary; WR: water runners.

### Immunohistochemistry of proximal convoluted tubule

**Fig 3:**
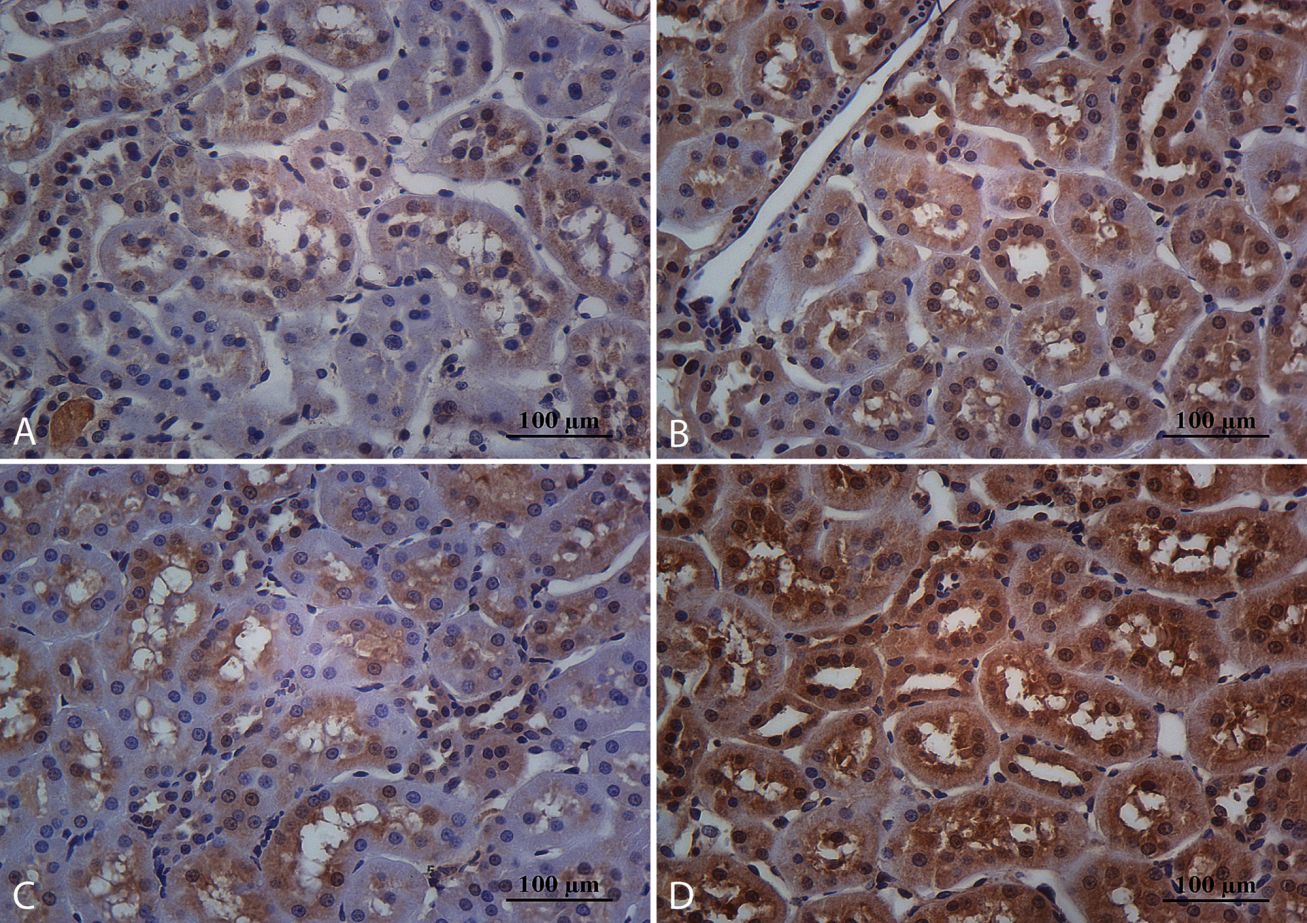
Immunohistochemical expression for trx1 at tubular level. Sedentary animals that consume cola soft drink (A) or water (C) showed lowest cytoplasmic staining that was partially reverted by aerobic training in CR (B) in comparison with WR (D). Magnification: 400X, scale bar 100 microns.

**Fig 4:**
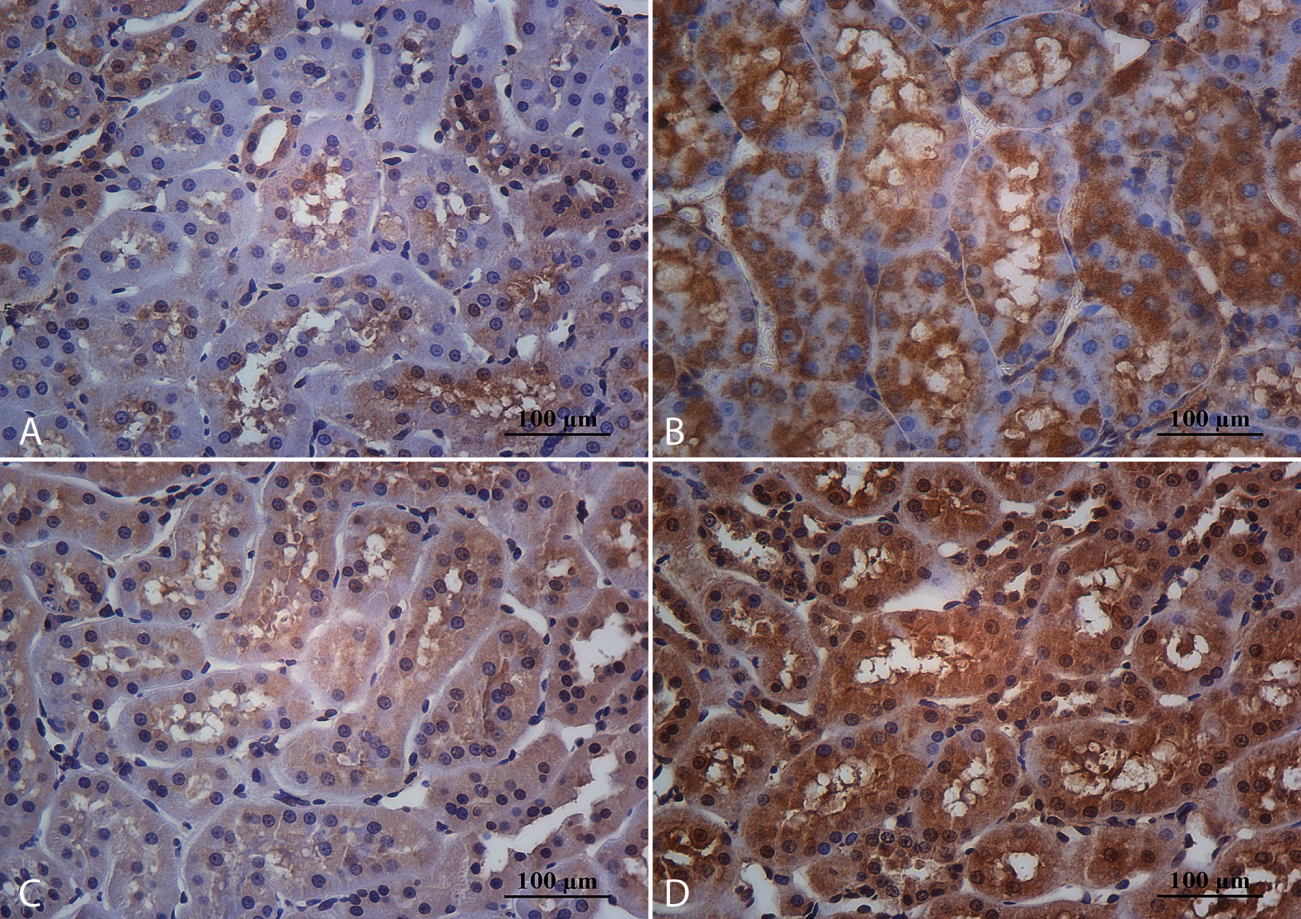
Immunohistochemical expression for prx1. The immunohistochemical profile for prx1 was similar than trx1 at the proximal tubule epithelia in CS (A), CR (B), WS (C) and WR (D) constituting a sensor for oxidative stress, favoring a protective effect in oxidative aggression. Magnification: 400X, scale bar 100 microns.

**Fig 5:**
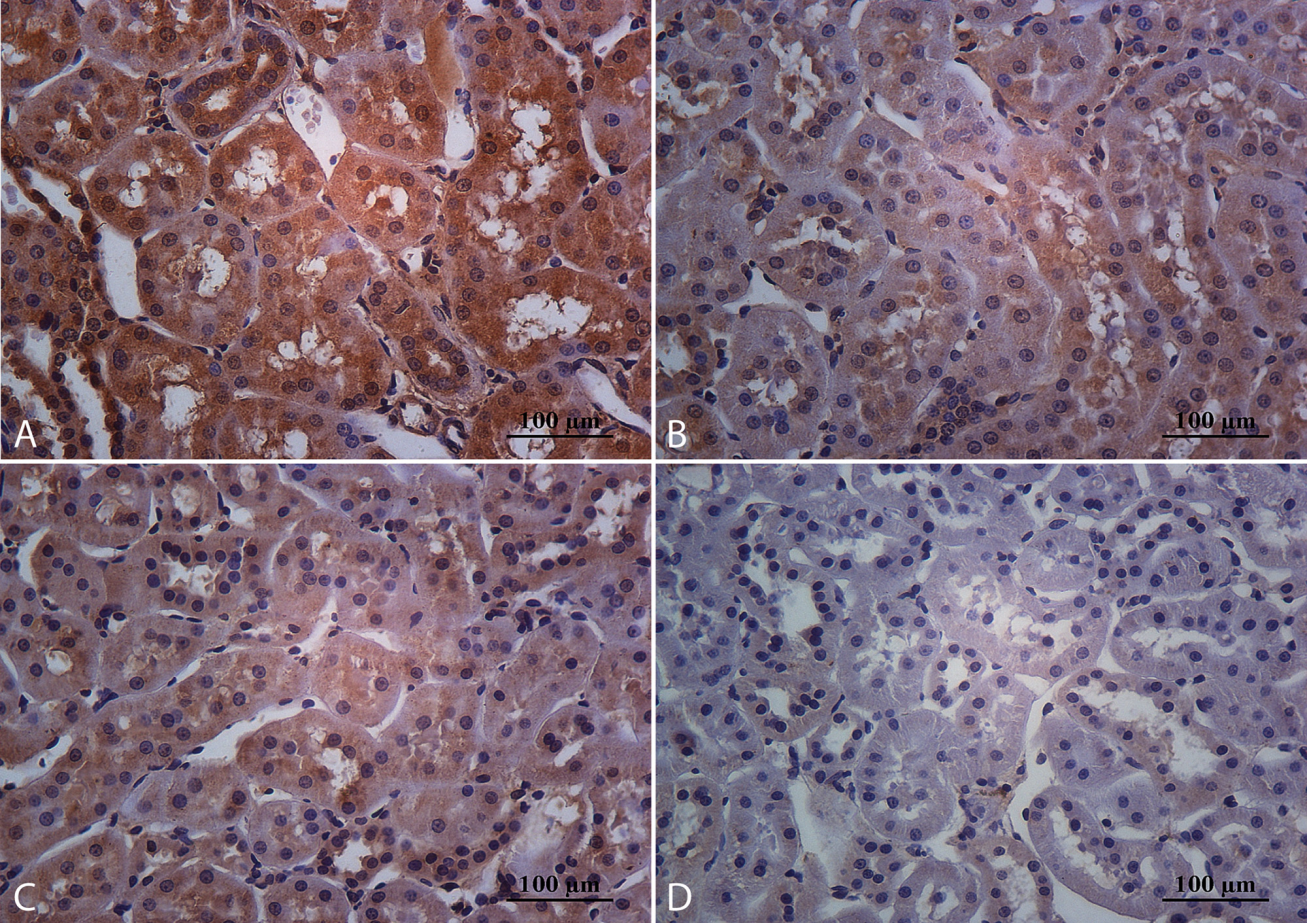
Immunohistochemical expression for IL6 at tubular level. Sedentary animals that consume cola soft drink (A) showed high cytoplasmic staining for IL6, that was reduced with aerobic exercise practice (B). The expression for this proinflammatory cytokine was lowest in experimental groups that drank water, both, sedentary (C) and runners’ (D). Magnification: 400X, scale bar 100 microns.

**Fig 6:**
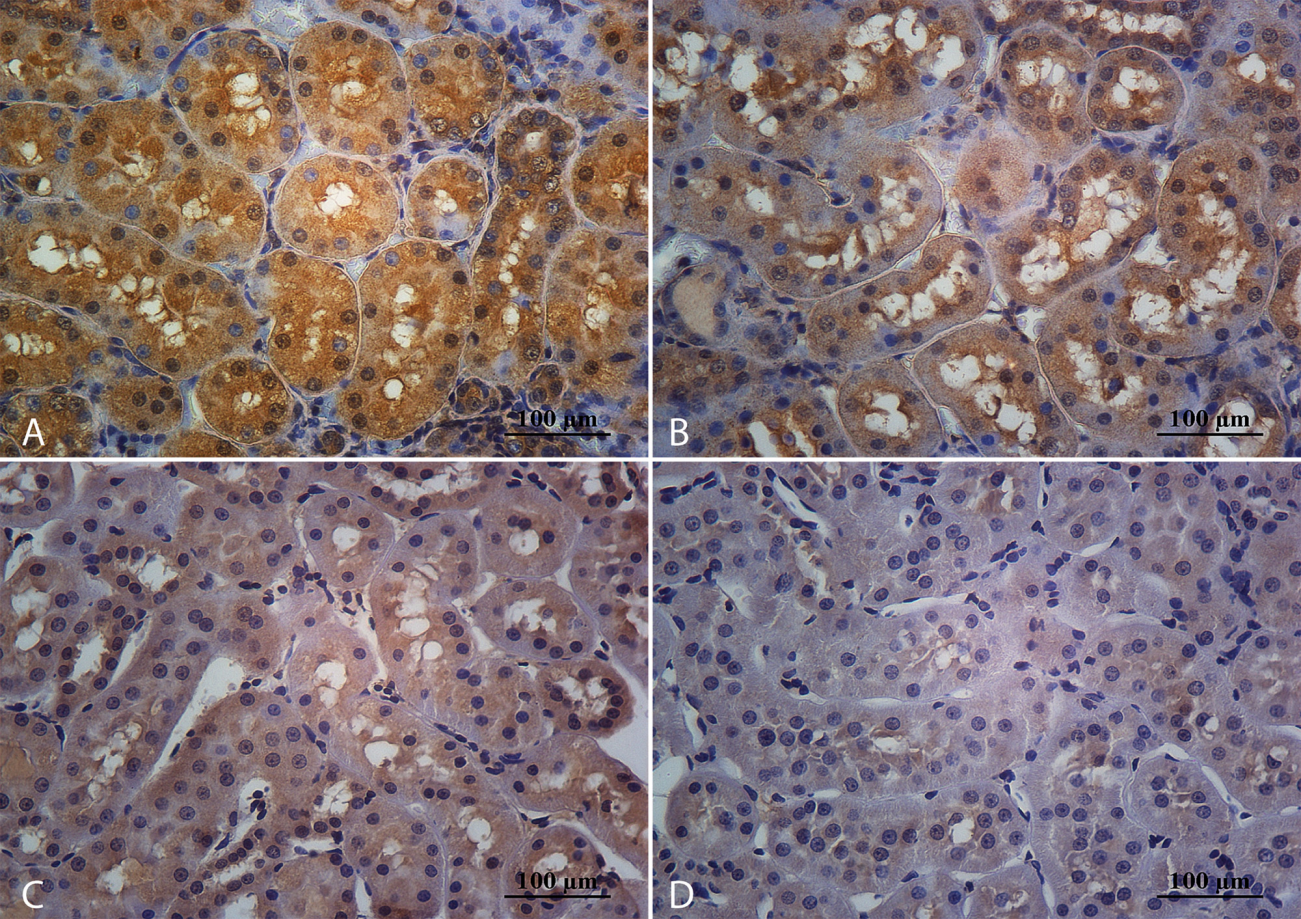
Immunohistochemical expression for TNFα. The immunohistochemical profile for this proinflammatory cytokine was similar than IL6 at tubular level in CS (A), CR (B), WS (C) and WR (D) groups. Magnification: 400X, scale bar 100 microns.

The sedentary rats showed a low cytoplasmic staining for trx1 (CS: 5.8±1.9 and WS: 6.9±4.5) and prx1 (CS: 6.5±2.2 and WS: 5.6±2.3) at the proximal convoluted tube compared to exercised animals (CR: 10.8±3.6 and 11.8±4.2; WR: 19.8±7.3 and 21.2±6 respectively) (Table 4, Figs 3 and 4). Chronic consumption of cola soft drink and sedentarism have favored the increased tubular expression for IL6 (CS: 25.5±7.7) and TNFα (CS: 18.3±7.9) (Table 4, Figs 5 and 6).

## Discussion

In the present study, chronic consumption of cola soft drink in adult rats was associated with increased levels of plasma TG, AIP values, histopathological score and glomerulosclerosis. Moreover, in coincidence with a previous report [5], cola drinking induced a net decrease in solid food intake with unchanged body weight.

Undoubtedly, high blood pressure is a recognized risk factor associated to renal damage. Therefore, a careful evaluation on blood pressure behavior is considered mandatory in any experimental study which investigates kidney disturbances, this in order to identify actual participation of arterial hypertension in renal lesions. In this sense, SBP was measured weekly during light cycle following the recommendations for the use of indirect methods for measuring SBP in animals [30, 31]. Notably, these authors showed similar results in SBP between telemetry and tail cuff method. Moreover, Soleimani M. and Alborzi P. [32] observed that fructose stimulates salt absorption in the intestine and kidney, thus high salt intake increases the fructose-induced hypertension. It is worth mentioning that a balanced commercial rat chow was used to feed the animals (0.2 g % sodium, normal composition for intake) in the present experiment. This condition might be a potential explanation to justify the behavior of SBP in the present study.

Chronic progressive nephropathy is a common and frequently recognized age-related renal disease of rat, in which incipient lesions are detectable as early as 2 month of age. A number of factors, primarily diet-related, have been shown to influence the incidence and severity of this spontaneous disease (dietary carbohydrate and fat, and serum lipids). In addition, oxidative stress potentially could exacerbate chronic progressive nephropathy [33]. It worth mentioning that in the present experiment, the age of *Wistar-Kyoto* rats was 16 weeks at the beginning of the study, and 40 weeks (10 months) at the end. The presence of glomerulosclerosis was estimated by point-counting and expressed in average percentage of glomerular area by the intrinsic characteristics of the method. Interestingly, Agarwal D et al., employing a similar procedure, showed glomerular lesions in their *Wistar-Kyoto* control group at 24 weeks of age [34]. In line with this, Kimura K et al., using a semiquantitative method, noted glomerular lesions in *Wistar-Kyoto* control group at 6 and 20 weeks of age [35].

In that study, the increase of sclerosis in glomeruli with age of animals suggests an age-related progression of chronic glomerular lesions in *Wistar-Kyoto* rats.

Applying the glomerular grade of these researches, our experiment shows a glomerular lesion grade 1 (< 25 or 30% of glomerular area affected), which is relatively low. In the same way, a histopathological score of 9.2±1.3% for WS group shows a mild compromise.

Although, the fasting time to 4-h may be an apparent limitation to interpret lipid results in the present study, it is noteworthy to mention that plasma TGs level show circadian rhythms [36] that can be disrupted by altering the dark phase of this chronobiological cycle [37]. Interestingly, a study in normal rats under a dark-light cycle and free access to normal chow showed that plasma triglyceride levels were 2-fold higher at midnight than those present at midday [38]. Furthermore, this cycling is coincident to the low activity in the light phase and high activity in the dark phase of the intestinal microsomal triglyceride transfer protein (MTP). The half-life of MTP is 4 days and the low diurnal activity reduces lipid absorption. Therefore, diurnal modulation of MTP is a major determinant of daily changes in plasma lipids. According to these data, and because in the present experiment, the rats were subjected to stressful conditions, especially in the exercise period, the fasting time (4-h), during a light phase where there is a diminution of plasma TGs levels (7:00 to 11:00 am) was chosen to determine TGs level.

Dyslipidemia may affect the kidney through systemic or local inflammation and oxidative stress, provoking nephrotoxicity as it has been reported [39]. This scenario is consistent with the increased histopathological score and glomerulosclerosis observed in CS group in the current study. Furthermore, TGs and AIP levels were increased in both groups that drank cola soft drink, although CR showed lower histopathological score than CS and a similar one to WR. These findings suggestting the moderate exercise improves renal damage regardless of the type of drink. The proximal tubule epithelial cells produce and release pro-inflammatory cytokines such as TNFα and IL6, this contributing to the extent of interstitial fibrosis, tubular atrophy, glomerulosclerosis, glomerular loss and CKD [40, 41]. The oxidative insult could facilitate this condition in sedentary animals that show a reduced cytoplasmic expression for trx1 and prx1, improved by aerobic exercise. It is recognized that the redox system plays an important role in protecting mammalian cells from reactive oxygen species [42, 43]. Previous reports indicate that simultaneous increase of trx1 and prx1 participates as a sensor of oxidative stress [44] such as we observed in our experimental aerobic training conditions. Likewise, aerobic exercise enhanced the nitric oxide production, favoring a protective adaptation by influencing on gene expression of antioxidant enzymes, with concomitant decline in oxidative aggression [45–47]. In this sense, both, cola soft drink consumption and sedentarism were two conditions that favored the increased tubular expression for IL6 and TNFα with relative reductions of trx1 and prx1, linked to higher histopathological scores. Interestingly, the elimination of both factors improved the morphological parameters in exercised animals that drank water. For example, smallest glomerular size associated with a mild increase in ΔCr at the end of the experiment shows the beneficial effects of lifestyle changes on kidney structure and function.

It is worth mentioning, those animals under aerobic exercise without soft drink consumption presented protective role related to Vg and ΔCr in the presented study. Since, the increased glomerular size is associated to glomerulosclerosis [48, 49], a potential mechanism, which may support the favorable effect of aerobic exercise observed in the current study, could be related to a suitable modulation in the glomerular size. In line with the experience of Joles et al [50], the results found in the group of sedentary rats in the current experiment suggest that hypertriglyceridemia may at least partially contribute to induce an additional percentage of glomerulosclerosis when compared with trained animals after chronic consumption of soft drink, this causing an impaired factor for glomerular integrity in this model. The largest glomerulosclerosis observed in CS may indicate glomerular susceptibility to hypertriglyceridemia. Interestingly, in this experiment, aerobic exercise training seems to moderate the negative effect generated by hypertriglyceridemia, this resulting in the glomerular injury avoidance. Certainly CS and CR groups display no differences in circulating TGs, this suggesting that both groups presenting equivalent potential factor for the development and progression of glomerulosclerosis. However, only the animals on aerobic exercise training did not show such morphology changes.

The heart rate ranges through the experiment were similar to published by others authors that used the same method to collect the SBP data [51]. From 3 months, CR showed an increase of heart rate compared with WR. Also, at 5 and 6 months, a higher heart rate was observed in CR than in CS. These findings suggest a progressive autonomic dysfunction due to sympathetic activation and vagal withdrawal by chronic consumption of cola soft drink. [52, 53]. On the other hand, from 3 months, WR showed a heart rate reduction compared with WS suggesting an increased vagal tonus due to the exercise practice [54].

In conclusion, the data presented in this study warns about the consequences of chronic consumption of cola drinks on lipid metabolism and especially regarding renal integrity that would be consistent with the increased risk of CKD observed in clinical studies. In addition, the outcomes presented in this study emphasize the protective role of exercise training on renal damage.

Limitation: a potential limitation of this study could be the fact that rats only drank cola soft drinks along the experiment. On the other hand, humans who drink excessive amounts of cola will probably consume other beverages. Furthermore, since cola is caffeinated, this particular condition may be associated with diuretic effect, this leading to fluids loss and consequently, chronic dehydration. Such condition may have influenced the health of rats in the cola group. However, this is unlikely as exercise improved health parameters.
